# Grain quality characteristics analysis and application on breeding of Yuenongsimiao, a high-yielding and disease-resistant rice variety

**DOI:** 10.1038/s41598-022-21030-9

**Published:** 2023-04-18

**Authors:** Zhanhua Lu, Zhiqiang Fang, Wei Liu, Dongbai Lu, Xiaofei Wang, Shiguang Wang, Jiao Xue, Xiuying He

**Affiliations:** 1 Rice Research Institute, Guangdong Academy of Agricultural Sciences, Guangzhou, 510640 China; 2 Guangdong Key Laboratory of New Technology in Rice Breeding, Guangzhou, 510640 China; 3 Guangdong Rice Engineering Laboratory, Guangzhou, 510640 China

**Keywords:** Plant breeding, Plant genetics

## Abstract

Rice quality is one of the main targets of rice breeding and is a complex trait that involves grain appearance, milling, cooking, eating and nutritional quality. For many years, rice breeding has contended with imbalances in rice yield, quality, and disease and lodging resistance. Here, the milling and appearance quality, cooking quality, starch rapid viscosity analyzer (RVA) profile, and nutritional quality of grains of Yuenongsimiao (YNSM), an *indica* rice variety with high yield, high quality and disease resistance, were determined. YNSM had excellent appearance and quality, with low amylose contents and high gel consistency, and these characteristics exhibited significant correlations with the RVA profile such as hot paste viscosity, cool paste viscosity, setback viscosity, and consistency. Moreover, 5 genes related to length-to-width ratio (LWR) as well as the *Wx* gene were used to detect the main quality genotype of YNSM. The results showed that YNSM is a semilong-grain rice with a relatively high brown rice rate, milled rice rate and head rice yield and low chalkiness. The results indicated that the LWR and food quality of YNSM might be related to *gs3*, *gw7* and *Wx*^*b*^. This study also reports the quality characteristics of hybrid rice developed using YNSM as a restorer line. The quality characteristics and the genotype for grain quality determined through gene analysis in YNSM may facilitate the breeding of new rice varieties that achieve a balance of grain yield, resistance and quality.

## Introduction

Rice (*Oryza sativa* L.) grain quality is one of the main targets of rice breeding. Rice grain quality involves several components, such as the grain appearance, milling, cooking, eating and nutritional quality^1–3^. In particular, cooking and eating quality traits, such as the amylose content (AC), grain thickness (GT), gel consistency (GC), pasting viscosity and aroma are important factors in determining the quality of cooked rice^4^.

Grain size is an important factor affecting rice yield and is also important for rice appearance quality. As a typical complex quantitative trait, grain size is closely associated with grain weight and usually measured by grain length (GL), grain width (GW), GT and length-to-width ratio (LWR)^5,6^. *GS3*, a major quantitative trait locus (QTL) for grain size, functions as a negative regulator of LWR and organ size^7,8^. *GS9* negatively regulates glume division by the GS9-OFP8/14-GSK2 pathway^6,9^. *GW5* negatively regulated rice GW^10^. *GW7/GL7* is a main QTL for grain length and width. The overexpression of *GW7* increases longitudinal cell division in the grain and reduces transverse cell division, resulting in more narrower grains and simultaneously improving rice yield and grain quality^11,12^. Additionally, *GW7* expression is directly regulated by OsSPL16/GW8, a transcription factor encoded by the grain-width locus *OsSPL16/GW8*^13^. However, LWR is closely related to milling quality parameters, such as the head rice yield.

Starch is the main chemical component of rice grains and is made up of amylose and amylopectin. The characteristics of starch directly affect the cooking and eating quality of rice^14^. AC has long been considered the most important factor in determining the quality of rice^15,16^. *Waxy*, the main gene that controls amylose synthesis, encodes granule-bound starch synthase (GBSS) and affects the AC in rice endosperm and pollen directly. In nonglutinous rice varieties, the *Wx* gene differentiates into *Wx*^*a*^ and *Wx*^*b*^ alleles; of these, wild rice and most *indica* rice had the *Wx*^*a*^ genotype, with high amylose content, the vast majority of *japonica* rice has the *Wx*^*b*^ genotype, with a relatively low amylose content^17,18^. Additionally, *Wx* can also control the gel consistency of rice^19^.

The starch viscosity profile, known as the RVA profile, refers to the curve of starch viscosity that varies with temperature during heating, a period of high temperature and cooling; the profile reveals the peak viscosity (PKV), hot paste viscosity (HPV), cool paste viscosity (CPV), peak time (PeT), paste temperature (PaT), breakdown viscosity (BDV, PKV minus HPV), consistency (CS, CPV minus HPV), and setback viscosity (SBV, PV minus PKV)^20,21^. It has been shown that the characteristic RVA profile values of rice starch are closely related to the rice cooking quality and food quality, especially BDV, CS and SBV^22^. Generally, the higher the amylose content is, the lower the BDV is, and the greater the SBV and CS are. The characteristic values of the RVA profile of rice starch can be used to distinguish differences in cooking and eating quality among rice varieties with similar apparent amylose contents.

Rice breeding in China is facing an imbalance among high yield quality, disease resistance and lodging. YNSM is a new rice variety that exhibits high yields, high quality and disease resistance that was bred by the Rice Research Institute of Guangdong Academy of Agricultural Sciences (GDAAS)^23–25^. To further reveal its quality characteristics, the milling quality, appearance quality, cooking and eating quality, nutritional quality, and RVA profile of rice starch were determined for YNSM. These results will provide a theoretical basis for the breeding of new rice varieties with high eating quality.

## Results

### Development of YNSM

**Figure 1:**
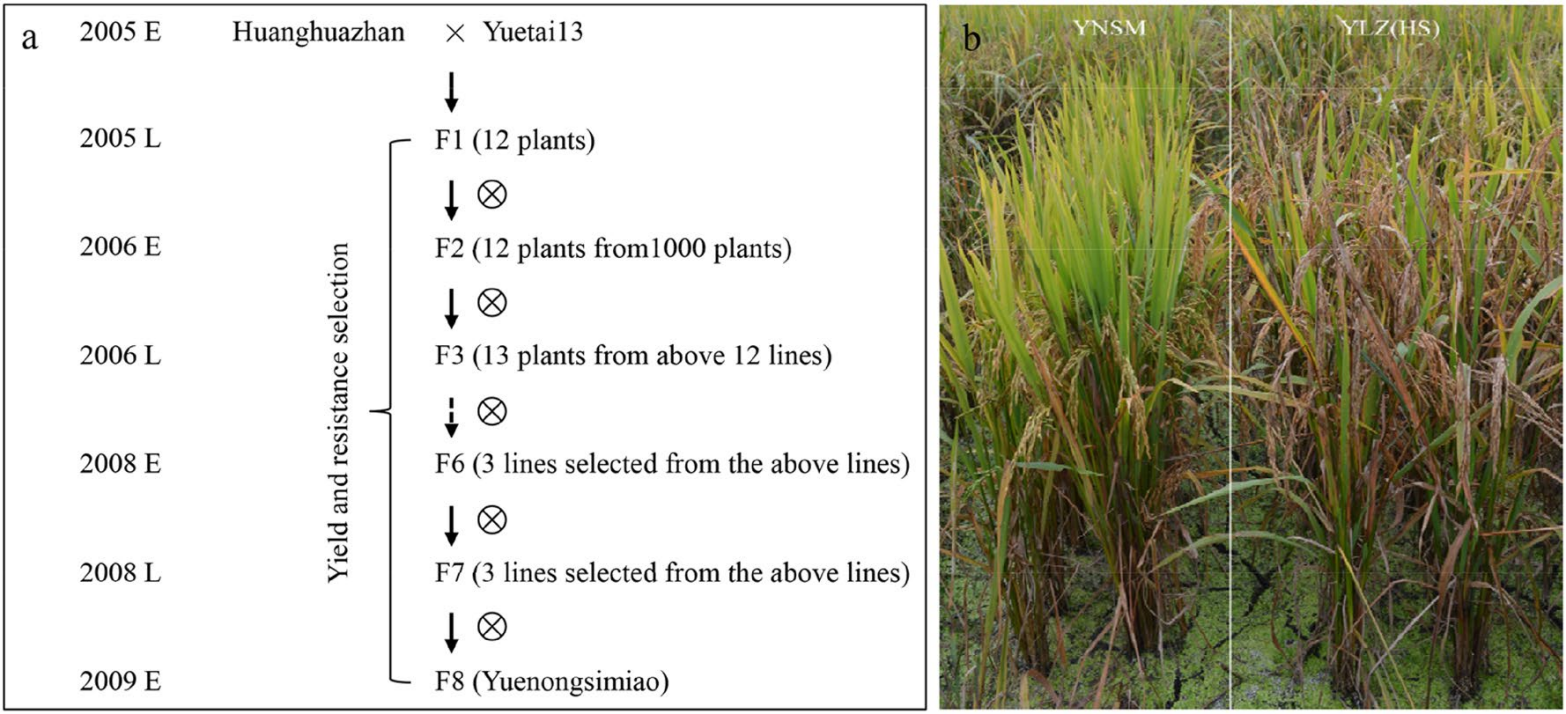
Flowchart and the blast resistance for the development of YNSM. (**a**), Flowchart for the development of YNSM; (**b**), The high blast resistance of YNSM. Yueluzhan (YLZ), a high susceptible (HS) variety to *Magnaporthe oryzae* in the diseased area in Conghua, Guangzhou.

YNSM was bred using Yuetai13, a high-yielding, disease-resistant variety, as the male parent and the good-quality variety Huanghuazhan as the female parent^26^. We used phenotypic selection (yield and plant height) for pedigree breeding from F_3_ generation^23^. In 2009, a line from the F_8_ generation with high-yielding and high resistance for blast was selected and named Yuenongsimiao, The blast resistance were detected in Conghua, Guangzhou. For its relative balance of high yield and blast resistance (Fig. 1), YNSM has been certified in Guangdong Province in 2011 (Guangdong authorized variety no.2011023), Hainan Province (2013), Jiangxi Province (2017) and Hubei Province (2017), China (http://www.ricedata.cn/variety/varis/610872.htm).

### The milling and appearance quality of YNSM

**Figure 2:**
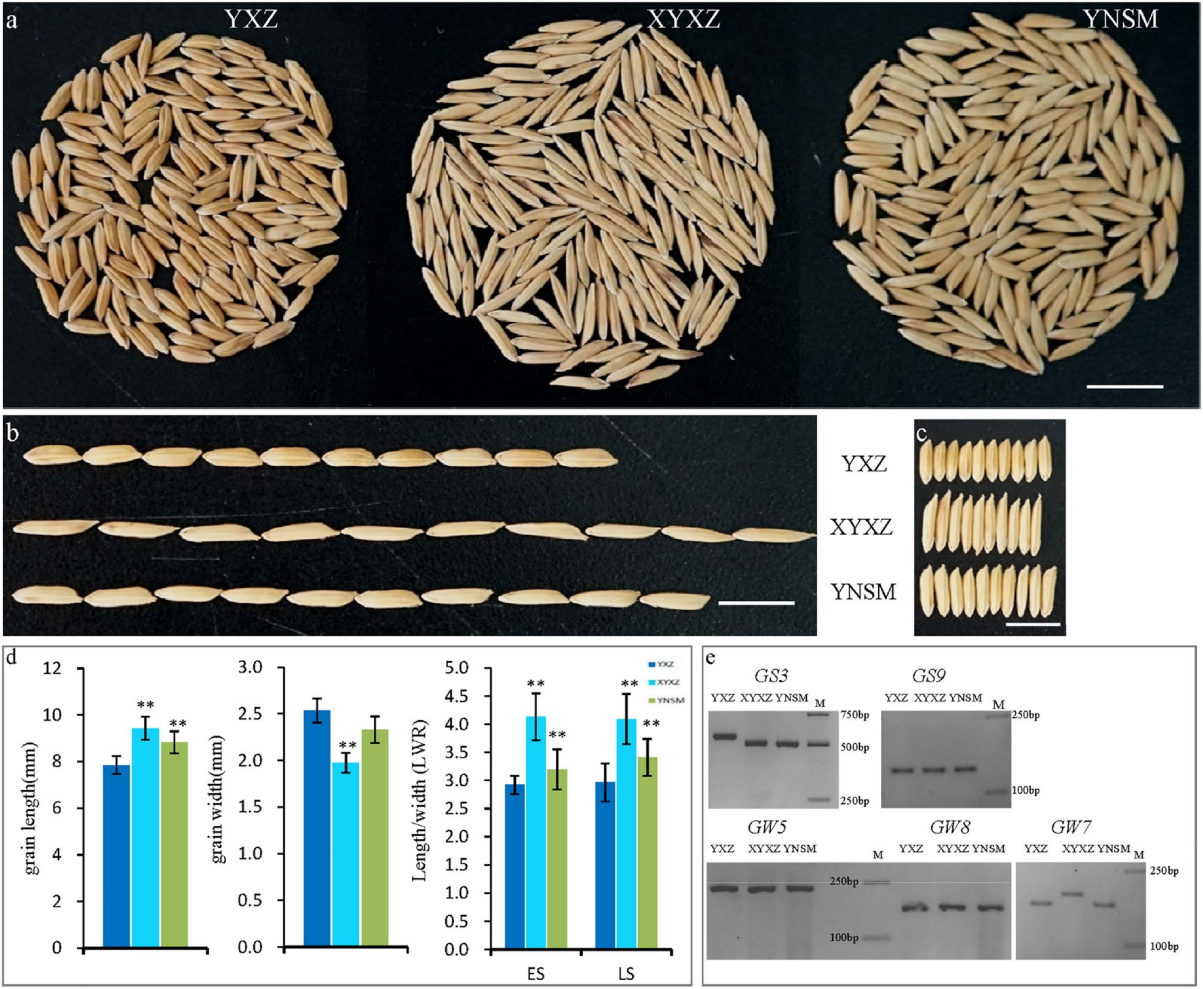
Grain appearance. (**a**), Rice grain appearance (n = 150 grains); (**b**), 10-grain length of 3 varieties; (**c**), 10-grain width of 3 varieties; (**d**), Statistical analysis of grain length, grain width and length/width ratio (LWR), ES, early season, LS, late season; (**e**), genotype of LWR genes. ES, early season; LS, late season; M, marker; Bar = 1 cm.

The grain size of YNSM is between those of Yuexiangzhan (YXZ) and Xiangyaxiangzhan (XYXZ). The grain length is 9.28 mm, the grain width is 2.3 mm, and the LWR is 3.2–3.4; these values are different from those of the small-grain variety YXZ and the thin long-grain variety XYXZ (Fig. 2a–d). Grain size is controlled mainly by genetic factors rather than environmental factors. To reveal the regulatory factors for YNSM LWR, we used known molecular markers. The results showed that LWR in YNSM, YXZ and XYXZ was controlled by *GS9, GW5,* and *GW8*. For the *GS3* gene, the long grain varieties YNSM and XYXZ had the genotype *gs3*, and the small variety YXZ had the genotype *GS3*. Moreover, unlike in YNSM and YXZ, LWR in XYXZ was found to be controlled by *GW7* (Figs. 2e, S1, S2).

**Figure 3:**
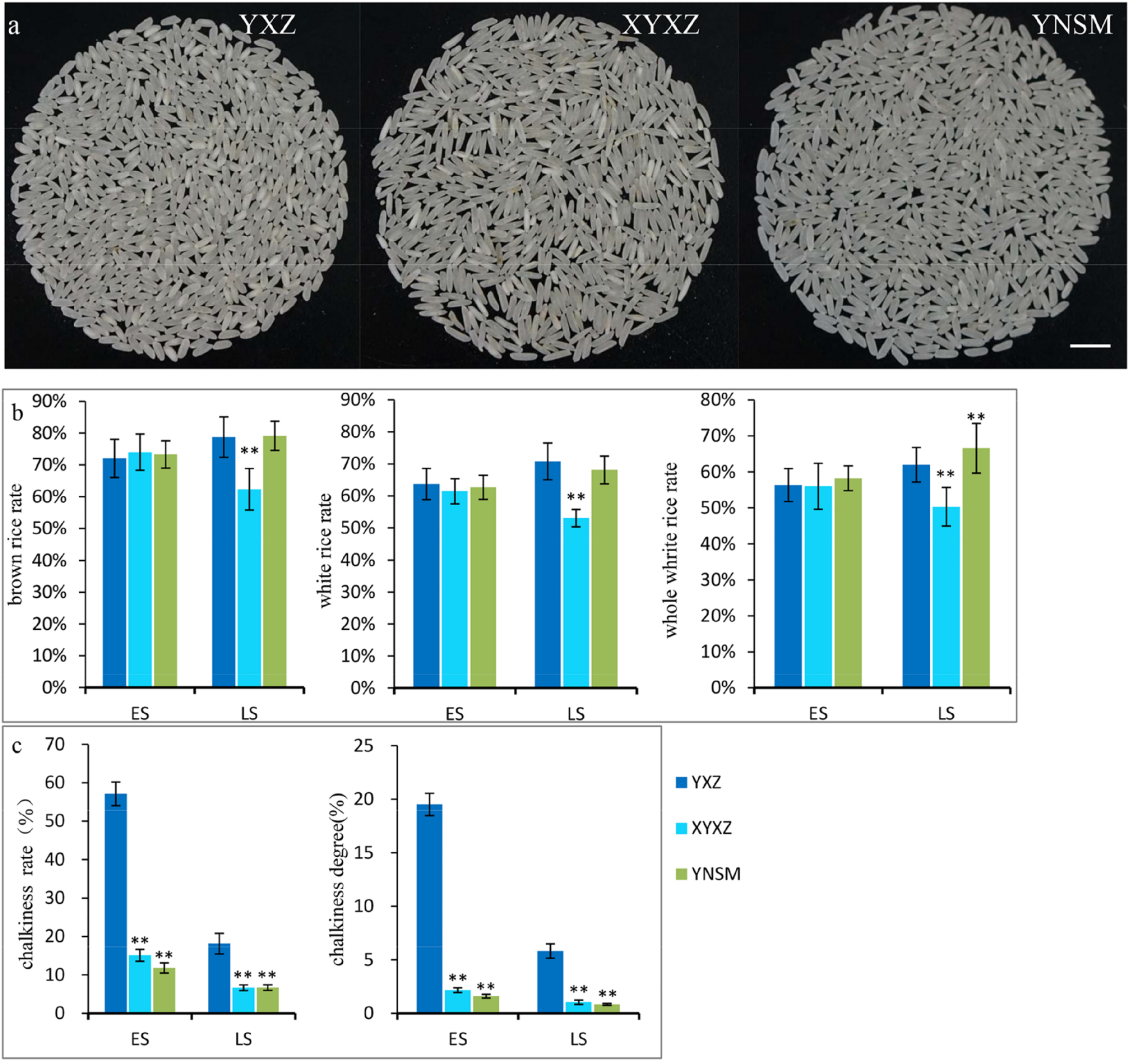
Milling quality analysis. (**a**), The appearance of milled rice; (**b**) and (**c**), Milling quality analysis. ES, early season, LS, late season. Bar = 1 cm. ES, early season; LS, late season.

The milling quality of the rice was different in different seasons. In the early season, the brown rice rate, milled rice rate and head rice yield of YNSM were similar to those of YXZ and XYXZ, at approximately 70%, 60% and 55%, respectively. However, in the late season, all of the milling quality parameters of YNSM were much better than those of YXZ and XYXZ (Fig. 3a,b).

Generally, the white grain rate and chalkiness rate were greatly affected by the season, and the rice quality was better in the late season than in the early season. The transparency level of YNSM was 3, which was better than those of YXZ and XYXZ. The chalkiness rate and chalkiness degree of YNSM in the early season were 11.84% and 1.61%, respectively. In the late season, the chalkiness rate and chalkiness degree were 6.71% and 0.84%, respectively, which were significantly lower than those of YXZ but equal to or better than those of XYXZ (Fig. 3c).

### Cooking quality characters of YNSM

The AC values of YNSM in the early and late seasons were 17.05% and 17.24%, respectively, which were lower than those of YXZ (25.05% and 26.47%) and similar to those of XYXZ (15.9% and 18.2%). The GC values of YNSM in the early and late seasons were 72.0 mm and 75.0 mm, respectively, which were similar to the values of XYXZ but significantly higher than those of YXZ (44 mm and 46 mm). These results showed that YNSM has equally low AC and high GC as other high-quality rice varieties. The eating score of YNSM (85.6–87.1/100) was similar to that of XYXZ (85.7–88.1/100) and significantly higher than that of XYZ (57.0–63.8/100) (Table 1).

**Table 1:** Primers used in this work.

Gene name	Primer name	Forward	Reverse	Fragment size (bp)	Tm (°C)	References
*Wx*	RA19	TACAAATAGCCACCCACACC	TTGCAGATGTTCTTCCTGATG	157	56	^37^
*GS3*	Chr301	TATTTATTGGCTTGATTTCCTGTG	GCTGGTTTTTTACTTTCATTTGCC	511	56	^9^
*GS9*	In0919	CGTTTAGGCTGGCTGC	CAGTTGGTGGTTTCGTAGAG	192	56	^9^
*GW7/GL7*	CHR701	AGGGCTGGGACTGAACTTTGT	ATGGACCCAGGCAAACACC	138	56	^9^
*GW5/qSW5*	CHR525	AAGAAAGCCCAAAACAACACA	CTTCCACCCTCAGTGTCGC	206	56	^9^
*GW8/OsSPL16*	GW8	AAAGAGACAGCCACGGAATC	ATCTTGAGATCCCACTCCAT	191/181	55	^38^

**Figure 4:**
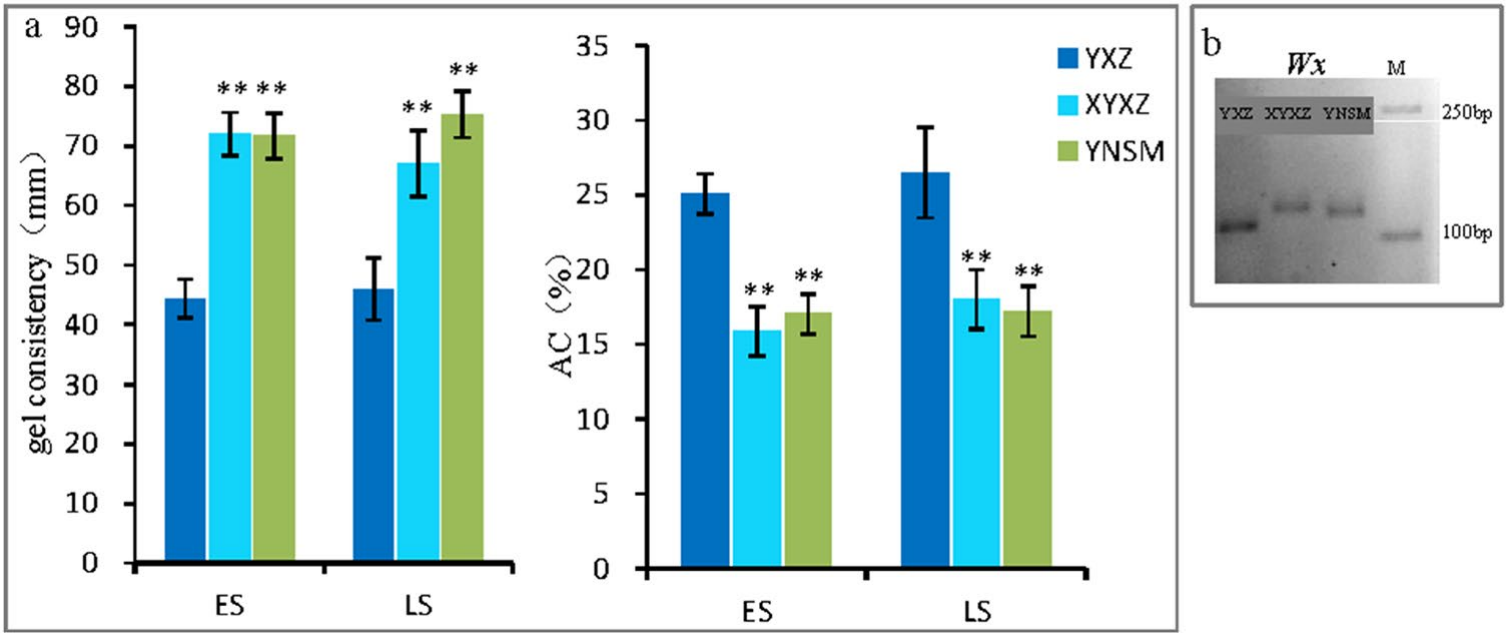
Cooking quality characteristics of YNSM. (**a**), GC and AC of the 3 tested rice varieties; (**b**), genotypes of cooking quality-controlled genes (*Wx*^*b*^). ES, early season; LS, late season.

Because *Wx* is the main gene that controls amylose synthesis, the genotype of *Wx* was determined in YNSM^17^. The results showed that YNSM and XYXZ had the *Wx*^*b*^ genotype, while YXZ had the *Wx*^*a*^ genotype, which was in line with the results for AC (Figs. 4, S1).

### The RVA profile of YNSM

In both the early and late seasons, the starch paste viscosity of the tested rice varieties displayed an increasing–decreasing-increasing trend. In the early season, the PV of YNSM was 248.9 RVU, which was half that of YXZ and similar to that of XYXZ (Table 2). The HV of YNSM was 88.1 RVU, which was significantly less than that of YXZ (312.1 RVU) but slightly higher than that of XYXZ (52.4 RVU). The CPV of YNSM was 163.9 RVU, which was substantially lower than that of YXZ (446.3 RVU) and higher than that of XYXZ (99.2 RVU). In the early season, the BD, CS and SB of YNSM were 160.8 RVU, -85.0 RVU and 75.8 RVU, respectively. These values were similar to those of XYXZ, but the BD was higher than that of YXZ, and the BD and CS were lower than those of YXZ. In the late season, the HPV, CPV and SB of YNSM were similar to those of XYXZ, but the PV, HPV and PeT were not significantly different from those of XYXZ. These results indicated that CPV, SB and CS were significantly correlated with taste quality.

**Table 2:** Starch-related physicochemical properties of the rice varieties used in this research.

Sample	PV (RVU)	HPU (RVU)	CPV (RVU)	BD (RVU)	SB (RVU)	CS (RVU)	PeT (min)	PaT (°C)
YXZ (ES)	426.9 ± 2.6	312.1 ± 4.7	446.3 ± 2.2	114.8 ± 2.1	134.2 ± 6.8	19.3 ± 4.8	6.0	72.3 ± 0.02
XYXZ (ES)	205.3 ± 2.3	52.4 ± 0.4	99.2 ± 0.1	152.9 ± 2.7	46.8 ± 0.3	−106.1 ± 2.4	4.4	74.0 ± 0.02
YNSM (ES)	248.9 ± 0.7	88.1 ± 1.0	163.9 ± 0.2	160.8 ± 0.3	75.8 ± 0.2	−85.0 ± 0.5	4.9	74 ± 0.01
YXZ (LS)	402.0 ± 1.2	260.0 ± 4.0	440.3 ± 6.8	142.0 ± 2.8	180.2 ± 10.8	38.2 ± 8.0	5.9	70.3 ± 0.4
XYXZ (LS)	431.1 ± 0.6	163.2 ± 4.9	275.2 ± 6.8	267.9 ± 5.5	112.0 ± 1.9	−155.9 ± 7.4	5.4	70.3 ± 0.5
YNSM (LS)	410.5 ± 3.5	185.0 ± 10.3	304.1 ± 13.6	225.5 ± 13.9	119.1 ± 3.3	−106.4 ± 17.2	5.7	70.3 ± 0.4

*ES* early season; *LS* late season.

Although the RVA profiles of the rice varieties were different between the early and late seasons, the correlations between profiles for the same variety were higher than 0.96, which means the RVA profiles of each variety in different seasons is positive correlation (Table 3). In the early season, the correlation between the RVA profiles of YNSM and XYXZ was 0.977, while it was lower than 0.77 between the profile of YXZ and that of YNSM or XYXZ. In the late season, the correlation between the RVA profiles of YNSM and XYXZ was 0.99, which was stronger than the correlation between the profile of YXZ and that of YNSM (0.887) or XYXZ (0.816). In general, rice quality tends to be higher in the late season than in the early season in South China; however, the relationship of the RVA profile of YNSM in the early season and the RVA profile of XYXZ in the late season was 0.994, further suggesting that the quality of YNSM is related to the high quality of XYXZ rather than YXZ.

**Table 3:** Relationships between RVA profiles in different seasons.

Sample	YXZ (ES)	XYXZ (ES)	YNSM (ES)	YXZ (LS)	XYXZ (LS)	YNSM (LS)
YXZ (ES)	1					
XYXZ (ES)	0.631	1				
YNSM (ES)	0.773**	0.977**	1			
YXZ (LS)	0.988***	0.646	0.791	1		
XYXZ (LS)	0.802**	0.961***	0.994***	0.816**	1	
YNSM (LS)	0.876**	0.919***	0.980***	0.887***	0.990**	1

*ES* early season; *LS* late season

***Significance level at *P* < 0.001; **Significance level at *P* < 0.01.

### Protein and fatty acid content of YNSM

**Figure 5:**
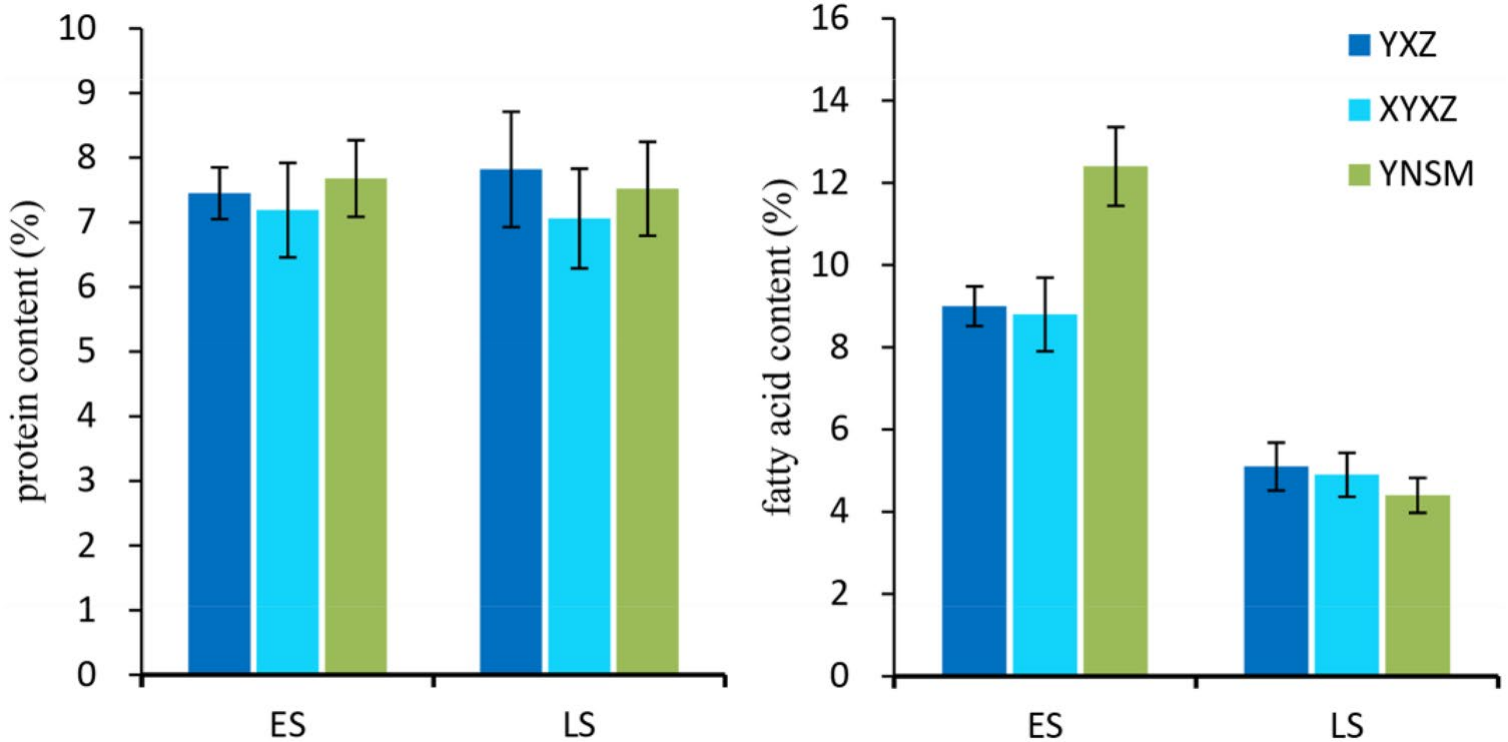
Protein and fatty content analysis. ES, early season; LS, late season.

Rice quality includes the nutritional quality of rice, such as its storage protein, fatty acid, anthocyanin and mineral contents^27,28^. Rice grains contain a certain proportion of protein and fat, which are related to its nutrition, luster and palatability. In the early season, YNSM had the highest protein and fat contents of the tested varieties; nonetheless, in the late season, there were no significant differences in protein and fat content among the rice varieties (Fig. 5). The protein contents of YNSM were 7.68% in the early season and 7.52% in the late season, which were similar to those of XYZ and higher than those of XYXZ. Interestingly, the content of fatty acids decreased greatly in the late season (Fig. 5).

### Quality-yield breeding applications of YNSM

YNSM not only produces excellent-quality rice but also exhibits outstanding comprehensive agronomic traits with highly resistant to blast and bacterial blight. YNSM is also an excellent restorer line (R1212) for two-line and three-line hybrid rice. At present, more than 20 hybrid rice combinations have been prepared with YNSM as a restorer line, and the rice quality of most of these hybrids is above grade 3, the national standard (Table 4, http://www.ricedata.cn/variety/). Among them, Guangtaiyou-YNSM, Jitianyou-YNSM, Huiliangyou-YNSM, Cliangyou-YNSM, Xinrongyou-YNSM, and Zaoyou-YNSM exhibited whole polished rice rates above 65%, GC values of 61–80 mm, AC values of 14.6–19.2%, and rice quality scores of to grade 2 . These results indicate that YNSM can improve the grain quality of hybrid rice.

**Table 4:** Quality characteristics of hybrid rice with YNSM as the restorer.

No	hybrid rice	CMS/PTGMS lines	quality level	head rice rate (%)	chalkiness rate (%)	chalkiness degree (%)	gel consistency (mm)	AC (%)	grain size (length/width)
1	Guang8you-YNSM	Guang8A	3	64.3	14	3.8	71	14.3	3.7
2	Hengfengyou-YNSM	HengfengA	3	63.6	9	2.4	65	4.8	3.5
3	Guangtaiyou-YNSM	GuangtaiA	2	65.4	7	1.6	61	15.2	3.3
4	Jitianyou-YNSM	JitianA	2	69.6	6	1.9	74	14.6	3.3
5	Changshengyou-YNSM	Changsheng843A	3	60.5	8	1.9	65	14.8	4
6	Taoyou-YNSM	Taonong1A	2	64.3	6	1.2	69	14.4	3.5
7	Huiliangyou-YNSM	1892S	2	67.9	10	2.8	80	14.6	3.4
8	Cliangyou-YNSM	C518S	2	68.4	8	2.7	72	15.2	3.2
9	Rongyou-YNSM	RongfengA	3	66.1	14	4.1	53	21.2	3.3
10	Xinrongyou-YNSM	XinrongA	2	70.1	9	2.3	75	14.4	2.8
11	Quanyou-YNSM	Quan9311A	3	65.2	18	4.7	79	15.2	3.1
12	Zaoyou-YNSM	ZaofengA	2	67.5	8	1.1	62	19.2	3.2
13	Long8you1212	Long8A	3	64.4	17	4.2	67	16.6	3.3
14	Jiuliangyou1212	33S	3	68.4	14	4.4	50	19.6	3.1
15	Longyou1212	Longxiang634A	3	65.2	20	4.9	72	14.3	3.5
16	Jingliangyou1212	Jin4128S	3	67.4	19	4.9	50	21.7	3.3
17	Longjingyou1212	Longjing4302A	3	61.1	16	3.1	52	15.7	3.4
18	Jingliangyou1212	Jing4155S	3	66.7	9.7	1.8	74	15.1	3.2
19	Longliangyou1212	Longke638S	2	60.1	4	0.4	62	15.3	3.2
20	Feiliangyou212	Longfei656S	3	57.6	9	2.2	51	17.7	3.3

## Discussion

### The influence of grain size on rice quality

Grain size is one of the most important factors influencing rice appearance quality and rice yield, and several genes related to grain size have been cloned^7,8,11–13^. *GS3* and *GW7* coordinately function in controlling grain size^7,13,29^. In this research, the genotype of the small-grain variety YXZ was *GS3/gw7*, that of YNSM was *gs3*/*gw7*, and that of XYXZ was *gs3/GW7*. Although *GW7* dramatically improved the appearance quality trait, it was also associated with lower head rice yield. Slender rice, such as XYXZ, is easily broken during milling, leading to secondary food losses. Therefore, rice yield and head rice yield should be considered in rice improvement with YNSM.

### Effect of starch content on taste

Amylose is the main component of grain starch. Usually, rice with more AC is not good for eating^21^. It has been clearly shown that *Wx* is the main gene that controls amylose synthesis^17^. For most rice varieties with good flavor, the genotype of *Wx* is *Wx*^*b*^*,* which causes low AC^17,18^. In this research, we revealed that the low AC is controlled by *Wx*^*b*^ in the YNSM genome, is useful information for the molecular breeding of high-quality rice (Fig. 4).

### Effects of RVA profile on taste

The characteristics of the rice starch RVA profile are an important factor affecting rice cooking and eating quality. The RVA profile can reflect not only the taste differences caused by differences in amylose content among different varieties but also the differences in taste and palatability that occur in rice varieties with similar amylose contents. In the RVA pasting viscosity parameters only BD, SB and CS seemed to be controlled by *Wx* gene, which is closed linked with good taste quality^21^. The RVA profile is also related to the gel consistency. Various rice varieties with low amylose contents usually have a high gel consistency, with higher BD and lower SB and CS^20^. In this research, YNSM and XYXZ were of similar quality, and their RVA profiles were markedly different from that of the high-AC rice variety YXZ. RVA profiles require few samples and have good repeatability; thus, they should be effectively applied in early generation selection. In this research, we found that the PaT was not significantly different among varieties but was significantly different in the different seasons, implying that the ripening temperature is important for rice quality^30^. Although YNSM and XYXZ both have low starch contents, there are still distinct differences in their taste quality, such as their nutrient quality content and RVA profile. Therefore, YNSM/XYXZ recombinant inbred lines (RILs) have been constructed to further reveal the genetic network of food quality.

### Improvements in rice varieties with high food and taste quality

The coordination of yield resistance and quality is an important goal of breeding. At present, given the large amount of genome-wide data, the continuous advancement of molecular marker-assisted selection and molecular design breeding, and CRISPR/Cas9 technology, it is possible to improve the yield, quality and resistance of rice in a precise and efficient manner. Here, we recommend YNSM, which not only produces high-quality grains but is also high yielding and disease resistance (Figs. 1,2; Tables 4; S1). It is known that *Pi2* is a broad-spectrum blast resistance gene and have been widely used in rice breeding^31^. In the previous research have revealed YNSM have *Pi2*^32^. In this research, we uncovered that *Wx*, *gs3*/*gs9*/*GW7* are important for the yield and quality. Hence, this study provided a new approach for rice breeding.

## Methods

### Materials

In this study, the *indica* rice variety YNSM (No: Yueshendao2011023) was tested, and the varieties Yuexiangzhan (YXZ, No: Yueshendao1998001), which has high ACC, and Xiangyaxiangzhan (XYXZ, No: Yueshendao2006044), which has low ACC and excellent flavor, were used as controls. All varieties were collected from Rice Research Institute of GDAAS, and planted at the experimental base of it in Guangzhou city (N 22°, E 112°), Guangdong Province, in the early season (planted in March) and late season (planted in July) of 2019. Before sowing, the seeds were accelerated for germination at 37 ℃, and planted at 3–4 leaf stage in the moderate fertility in the moderate-fertility soil. All varieties were planted for 3 times. Fertilization and weeding of plants in the present study complies with international, national and/or institutional guidelines.

### Rice quality determination

Seeds were harvested after maturity, and 100 g of air-dried rice that was intact and free from pests and diseases was stored in a dry, ventilated indoor environment (25 ℃) for 3 months until the physicochemical properties of the seeds stabilized^33^. Before testing, the samples to be tested were placed in a dry, ventilated place or an air-conditioned room for approximately 7 days to keep the moisture content of the samples below 14%.

### Processing quality determination

An amount of 30 g of rice grain was taken from each of the samples. Using a rice huller (JLG-III), the sample was processed into brown rice, placed into a brown rice polishing machine (JNM-III) and processed. Then, the rice was polished to a diameter of 1.5 mm and passed through round holes in a screen to obtain milled rice. Finally, the samples were sieved and isolated to obtain whole white rice. Brown rice rate = brown rice weight (g)/30 (g) × 100%, milled rice rate = white rice weight (g)/30 (g) × 100%, head rice yield = whole white rice weight (g)/30 (g) × 100%.

### Appearance quality determination

An SC-E rice appearance quality detector (Wanshen Testing Technology Co. LTD, Hangzhou, China) was used to measure the length/width ratio of whole brown rice and the chalky grain rate, chalkiness degree and transparency of milled rice. Finally, all the refined rice was ground with a miniature universal pulverizer and screened with a 100-mesh sieve. The rice flour was stored at 4 °C for physical and chemical property analysis.

### Evaluation of rice cooking and eating quality

The AC and GC were measured using standard procedures as described previously. The AC was determined using a colorimetric method with KI-I_2_ and measured by a continuous flow analyzer (Futura-II) with the manufacturer’s protocol; GC was measured as the gel length, with longer gels being considered softer than shorter gels^34^. The paste viscosity of rice starch was measured using a RVA profile by RVA-TecMaster (Perten, Sweden). Briefly, 3 g of rice flour was mixed with 25 mL of water in the RVA sample can. An RVA-Super 3 Viscometer instrument operated using Thermocline Windows control and analysis software version 1.2 was used (Newport Scientific, Sydney, Australia). The RVA profile is generally composed of five primary and two secondary parameters of the pasting curve: PKV, HPV, CPV, PeT, PaT, BDV, CS, and SBV.

The grain protein content in rice flour was determined by the Kjeldahl method using a Kjeltec 2300 Autoanalyser (Foss AB, Sweden). A nitrogen conversion factor of 6.25 was used to calculate the grain protein content^35^.

The taste value of the rice was measured by a SATAKE rice taste analyzer^36^. Each rice sample was washed with water 3 times in a cup with filtered water. Then, water was added to the stewed cup at a rice:water ratio of 1:1.4. After the rice was cooked and cooled, 7 ± 1 g of milled rice was weighed out, pressed with a molding tool into a microcake shape, and then put into a rice taste tester to determine the rice taste value.

The protein content of the brown rice was determined according to NY/T3-1982, and the fatty acid content of the brown rice was determined according to GB/T5510-2017. The fatty acid content was expressed as the potassium hydroxide mass (mg) needed to neutralize the free fatty acids in a 100 g sample.

### PCR-based genotyping

Plant genomic DNA was extracted from the fresh leaves of heading-stage plants using a previously described CTAB protocol^23^. The molecular markers for genotyping *Wx*, *GS3*, *GS9*, *GW5*, *GW7* and *GW8* are listed in Table 1. PCR amplification was performed on a Bio-Rad C1000 Touch Thermal Cycler (USA), and the protocol for PCR amplification with the appropriate parameters was performed as described previously. The PCR products were resolved on a 3.0% agarose gel in 1 × TBE buffer^23^.

### Statistical analysis

Statistical analysis was performed with independent samples using Student’s *t-*test and analysis of variance (ANOVA) and report the least Significant differences (LSD). The data are represented as means ± standard deviations (means ± SDs).

## Conclusion

Rice quality is one of the main targets of rice breeding and is a complex trait involving grain appearance, milling, cooking, eating and nutritional quality. For a long time, rice breeding has experienced imbalances among yield, quality, and disease and lodging resistance. This study showed that the *indica* rice variety YNSM has excellent quality, with a relatively high brown rice rate, milled rice rate and head rice yield, low chalkiness and amylose content, and long gel consistency and that these properties were significantly correlated with the HPU, CPV, SB, and CS. This study also revealed that the grain size and food quality of YNSM may be related to *gs3*, *gw7* and *Wx*^*b*^. This study also reported the quality characteristics of hybrid rice developed using YNSM as a restorer line. These results will facilitate the improvement of new rice varieties that achieve a balance of grain yield, resistance and quality.

## Supplementary Information

Supplementary Information.

## Abbreviations

AC: Amylose content
GL: Grain length
GW: Grain width
LWR: Length-to-width ratio
RVA: Rapid viscosity analyzer
GC: Gel consistency
HPV: Hot paste viscosity
CPV: Cool paste viscosity
PKV: Peak viscosity
SBV, PV minus PKV: Setback viscosity
CS, CPV minus HPV: Consistency
BDV, PKV minus HPV: Breakdown viscosity
PeT: Peak time
PaT: Paste temperature
