# Supplementary material for: Grain quality characteristics analysis and application on breeding of Yuenongsimiao, a high-yielding and disease-resistant rice variety
Source: Sci Rep. 2023 Apr 18;13:6335. doi: 10.1038/s41598-022-21030-9 (PMC10113224; doi:10.1038/s41598-022-21030-9)
Supplement: Supplementary file 1 — Supplementary Information. [file 41598_2022_21030_MOESM1_ESM.docx]

**Grain quality characteristics analysis and application on breeding of Yuenongsimiao,** **a high-yielding and disease-resistant rice variety**

Zhanhua Lu^1,2,3#^, Zhiqiang Fang^1,2,3#^, Wei Liu^1,2,3^, Dongbai Lu^1,2,3^, Xiaofei Wang^1,2,3^, Shiguang Wang^1,2,3^, Jiao Xue^1,2,3^, Xiuying He^1,2,3^*

^1^ Rice Research Institute, Guangdong Academy of Agricultural Sciences, Guangzhou 510640, China

^2^ Guangdong Key Laboratory of New Technology in Rice Breeding, Guangzhou 510640, China

^3^ Guangdong Rice Engineering Laboratory, Guangzhou 510640, China

^#^ These authors contributed equally to this work.

*Corresponding author (e-mail): [hexiuying@gdaas.cn](mailto:hexiuying@gdaas.cn)

**Supplementary information**

Fig S1 genotype detection of Waxy gene (*Wx^b^*) LWR genes (*GW7, GS3 and GS9*) in YXZ, XYXZ and YNSM. M, marker. Red boxes regions are used for the main Figure (Fig 2e,4b)


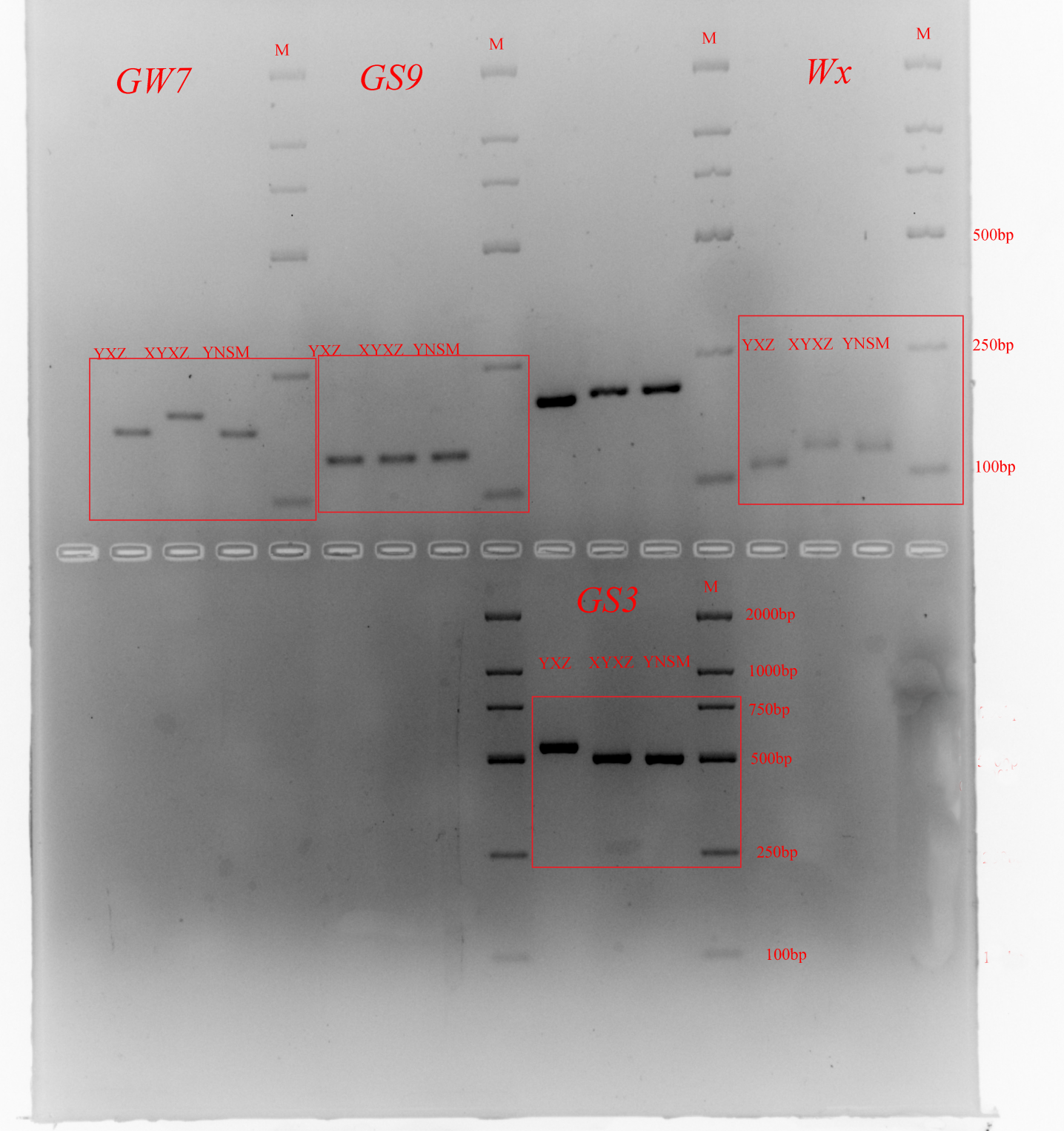


Fig S2 genotype detection of LWR genes (*GW5* and *GW8*) in YXZ, XYXZ and YNSM. M, marker. Red boxes regions are used for the main Figure (Fig 2e)


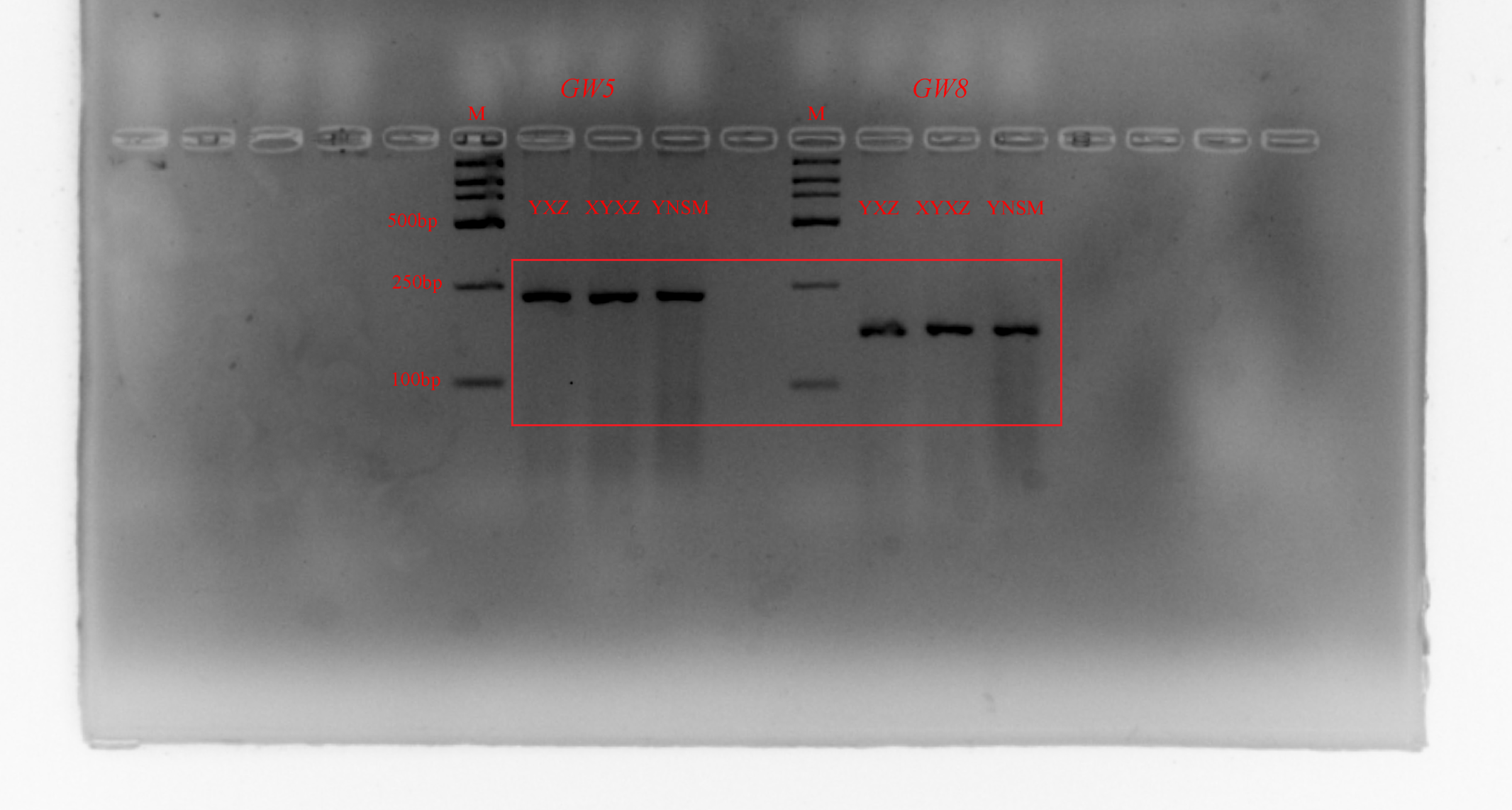


Table S1 Regional test results of YNSM

| **Main agronomic trait** | **Guangdong Province (2011)** | **Hainan Province**  **(2013)** | **Guangxi Province**  **(2017)** |
| --- | --- | --- | --- |
| **Whole growth period (d)** | 112.0 | 122.0 | 120.6 |
| **Plant height (cm)** | 97.5 | 95.5 | 102.0 |
| **Panicle number/ 667 m^2^** | 196000 | 1989000 | 176000 |
| **Total number per panicle** | 123.0 | 128.7 | 136.2 |
| **Seed setting rate** | 87.6 | 84.4 | 88.3 |
| **1000-grain weight** | 22.3 | 20.9 | 23.4 |
| **Quality** | GB II | / | GB III |
| **CK** | Jingxian 89 | Texianzhan 25 | Liushayouzhan 202 |
| **Yield (Kg/667 m^2^)** | 404.3 | 421.5 | 453.7 |
| **Compared with CK ± (%)** | 2.1 | 3.4 | 10.2 |
